# Comparison and Discrimination of Two Major Monocultivar Extra Virgin Olive Oils in the Southern Region of Peloponnese, According to Specific Compositional/Traceability Markers

**DOI:** 10.3390/foods9020155

**Published:** 2020-02-06

**Authors:** Vasiliki Skiada, Panagiotis Tsarouhas, Theodoros Varzakas

**Affiliations:** 1 Department of Food Science and Technology, University of Peloponnese, Antikalamos, 24100 Kalamata, Greece; 2 Department of Supply Chain Management (Logistics), International Hellenic University, Kanellopoulou 2, 60100 Katerini, Greece; ptsarouhas@ihu.gr

**Keywords:** EVOO, cv. Koroneiki, cv. Mastoides, south Peloponnese, Greece, fatty acids, sterols, botanical origin

## Abstract

The qualitative characteristics and chemical parameters were determined for 112 virgin olive oil samples of the two dominant olive cultivars in the southern region of Peloponnese, cv. Koroneiki and cv. Mastoides. As no relevant data exist for this geographical area, yet one of the most important olive-growing regions in Greece, this study aimed to evaluate and evidence the differences on specific chemical characteristics of the oils because of their botanical origin. Olive oils of Koroneiki variety were characterized by a three-fold lower concentration in heptadecanoic and heptadecenoic acid compared to oils of cv. Mastoides. In addition, Mastoides oils exhibited higher β-sitosterol and total sterols concentration and lower Δ-5-avenasterol and total erythodiol content compared to Koroneiki olive oils Analysis of variance and principal component analysis of the GC-analyzed olive oil samples showed substantial compositional differences in the fatty acid and sterolic profile between Koroneiki and Mastoides cultivars. Hence, results demonstrate that the fatty acid and sterolic profile can be used as exceptional compositional marker for olive oil authenticity.

## 1. Introduction

Nowadays, the increased awareness regarding the beneficial impact and nutritional properties of extra virgin olive oil (EVOO) is a key factor which has led to a higher demand on international olive oil consumption [1,2,3]. On the other hand, the increased globalized world and higher cost of olive oil production compared to other vegetable oil sources has led to adulteration with cheaper oils of lower grade. Consequently, a controlled traceability system has become a requirement in the olive oil supply in order to protect consumers against any unapproved and fraudulent practices. Thus, olive oil authenticity and traceability are crucial in order to overcome frauds in the international olive oil trade [4,5]. For this reason, the European Union has adopted a series of regulations in order to certify, protect, and guarantee the quality of the monovarietal olive oils [6,7,8,9,10]. The quality of these monovarietal olive oils is associated with specific characteristics directly related to the olive cultivar [11,12]. Therefore, the authenticity efforts are concentrated on the identification of their botanical origin as well as their adulteration with lower quality or less costly cultivars of lower commercial value.

The production of monovarietal olive oils has increased at a great extent lately since the quality of an olive oil depends on the olive variety from which it originates. Nowadays, several efforts have focused on the investigation of one or several compounds present in olive oils to differentiate olive varieties. Compositional markers include major and minor components providing useful information on olive cultivars to differentiate their botanical origin [11].

Despite the fact that in Greece the number of autochthonous monocultivars is greater than 40, with the most common olive cultivar (for olive oil production) being cv. Koroneiki, the majority of the other autochthonous cultivars remain poorly investigated. Olive cultivation is greatly spread in central Greece, with almost 40% of olive production being centered in the Peloponnese region [13,14]. In southern Peloponnese, among the predominant monovarietal olive oils cultivated are cv. Koroneiki and cv. Mastoides [15]. Koroneiki is the most well-known and systematically cultivated variety, the name of which derives from Koroni, a small village located southeast of Messinia in Peloponnese. On the other hand, Mastoides (referred to locally as Athinolia) is less exploited and cultivated in specific areas of Peloponnese mainly in south Lakonia, Argolida as well as in western Crete. According to our knowledge, there are only two publications for cv. Mastoides, performed in the island of Crete, by Stefanoudaki et al. focusing on the potential of triglyceride and fatty acid composition data as indicators of geographical and botanical origin [16,17].

The present work focuses on the evaluation and characterization of the performances of the two dominant and autochthonous monovarietal olive oils from cv. Koroneiki and cv. Mastoides, cultivated in the south of Peloponnese based on their qualitative and chemical characteristics. Emphasis was given on the influence of cultivar on their fatty acid and sterolic profile in order to be used as compositional/traceability markers in terms of their botanical origin.

## 2. Materials and Methods

### 2.1. Geographical Distribution, Sampling, and Sample Maintenance

A total of one hundred and twelve (N = 112) olive oil samples were collected during the harvesting period 2014–2015 from two neighborhood regions in the southern region of Peloponnese in Greece. In particular, sixty nine (69) olive oil samples of Koroneiki cultivar originated from the region of Messinia and forty three (43) olive oil samples of Mastoides cultivar from the southeast part of Lakonia. Both regions are characterized by similar climatic conditions. Olive fruits were picked at the optimal stage of maturity. Samples were transferred to local oil mills in solid, vented, food-grade harvest bins or in suitable harvesting bags. Olive fruits were processed within 24 h, and the same post-harvest conditions were maintained at all cases. In detail, the leaves were removed from the olive fruits, washed and then sent to the crusher. Malaxation was carried out at low temperatures (27–28 °C) for 30–40 min. The obtained olive paste was decanted (horizontal centrifuge) and the resulting olive oil was vertically centrifuged. Olive oil samples were stored directly in 1 L air-tight dark-green glass bottles at 4 °C until further analysis. Quality parameters were analyzed in triplicate, while all the other examined chemical parameters were determined in duplicate.

### 2.2. Chemicals and Standards

*All* solvents used for the determination of spectroscopic indices (K_232_, K_268_), free fatty acid and peroxide value were purchased from Sigma (St. Louis, MO, USA). The internal standard, 5a-cholestan-3β-ol and the fatty acid methyl esters (FAME) standard mixture were purchased from Sigma (St. Louis, MO, USA). Silica gel plate for thin-layer chromatography was purchased from Fluka (Buchs, Switzerland) and the silylation reagents, pyridine, hexamethyldisilizane, and tri-methylchlorosilane were purchased from Supelco (Bellefonte, PA, USA). Acetone, methanol, n-heptane, chloroform and diethyl-ether were purchased from Sigma (St. Louis, MO, USA).

### 2.3. Determination of the Physicochemical Quality Parameters

Free fatty acid, peroxide value and spectroscopic indices (K_232_ and K_268_) were carried out, following the analytical methods described in the Regulation EEC/2568/91 of the European Commission and later amendments [18]. Free fatty acid was expressed as the percentage of oleic acid and peroxide value was given as milliequivalents of active oxygen per kilogram of oil (meq O_2_ kg^−1^). K_232_ and K_268_ extinction coefficients were calculated from absorption at 232 and 268 nm respectively. These absorptions are expressed as specific extinctions E (the extinction of 1% *w*/*v* solution of the oil in isooctane, in a 10 mm cell) conventionally indicated by K “extinction coefficient”.

### 2.4. Determination of Sterols and Triterpene Dialcohols

The individual sterols, total sterols, and triterpene dialcohols were determined according to the method adopted by EEC/2568/91 regulation, Annexes V with later amendments [18]. The oil sample, with added 5a-cholestan-3β-ol, as an internal standard, was saponified with potassium hydroxide in ethanolic solution and the unsaponifiable matter was extracted with diethyl ether. The sterol and triterpene dialcohol fractions were separated from the unsaponifiable matter by thin-layer chromatography on a basic silica gel plate. The fractions recovered from the silica gel were transformed into trimethylsilyl ethers (TMSE) by the addition of pyridine-hexamethyldisilizane-tri-methylchlorosilane (9:3:1, *v*/*v*/*v*). Sterols (%) and triterpene dialcohol contents were determined with a Shimadzu (GC-2010) gas chromatograph equipped with a flame ionization detector (FID), a DB-5 (30 m × 0.32 mm × 0.25 μm) capillary column and an autosampler injector. The operating conditions were as follows: injection temperature 280 °C, column temperature 265 °C, detector temperature 310 °C, splitting ratio (1:50), flow rate 1.4 mL/min, and injection volume of 1 μL of TMSE solution. Individual sterols were identified based on their relative retention times with respect to the internal standard, 5a-cholestan-3β-ol, according to the standardized reference method [18]. The sterols and triterpene dialcohols eluted in the following order: cholesterol, 24-methylen-cholesterol, campesterol, campestanol, stigmasterol, Δ7-campesterol, Δ5,23-stigmastadienol, clerosterol, β-sitosterol, sitostanol, Δ5-avenasterol, Δ5,24-stigmastadienol, Δ7-stigmastenol, Δ7-avenasterol, erythrodiol, and uvaol (calculated as total erythrodiol). Sterol and triterpene diol concentrations were calculated as mg/kg of oil with respect to the internal standard. Results were expressed as proportions (%) of total sterols. The sum of Δ5,23-stigmastadienol, clerosterol, β-sitosterol, sitostanol, Δ5-avenasterol, and Δ5,24-stigmastadienol represents apparent b-sitosterol. Mean values of duplicate experiments in each sample were used for further statistical analysis.

### 2.5. Determination of Fatty Acid Composition

The fatty acid composition was determined according to the official method of the Regulation EEC/2568/91, Annex IV with amendments [18]. The fatty acid methyl esters (FAME) were obtained by cold alkaline transesterification with methanolic potassium hydroxide solution and extracted with *n*-heptane. FAME were analyzed on a model GC-2010 Shimadzu chromatograph, equipped with a BPX-70, (60 m × 0.25 mm × 0.25 μm), capillary column and a flame ionization detector (FID). The carrier gas was helium, with a flow of 1.5 mL/min. The temperatures of the injector and detector were set at 250 and 260 °C respectively and the oven temperature was increased gradually from 165 to 225 °C in 35 min. The injection volume was 1 μL. Quantification was achieved using a FAME standard mixture. The results were expressed as a percentage of individual fatty acids.

### 2.6. Statistical and Chemometric Analysis

Results were expressed as mean values ± standard deviation (SD). Data was processed with MINITAB 18 software. Thus, it was possible to obtain the minimum and the maximum value of the sample, mean, and standard deviation (SD). Τhe minimum and the maximum value of the sample are the values of the largest and smallest elements of a sample. In statistics, the difference between the largest and smallest values (range) provides an indication of statistical dispersion. Differences between means were tested for statistical significance using analysis of variance (ANOVA). Statistical significance level was set at *p* < 0.05. In addition, principal component analysis (PCA) was applied to study the relations between the two (2) mono-cultivars (cv. Koroneiki vs cv. Mastoides) on the examined chemical properties.

## 3. Results and Discussion

### 3.1. Physico-Chemical Parameter of the Two Major Olive Cultivars of Southern Peloponnese

It is well established that the quality parameters of olive oil are mainly altered by factors causing injuries to the olive fruits such as olivefly attacks, improper methods during olive harvesting as well as poor post-extraction conditions (e.g., inappropriate storage and packaging) [19]. Free fatty acid, peroxide value and spectrophotometric absorption were examined in the studied olive oils. Mean values for each analytical parameter, as well as minimum and maximum values of the measured parameters are reported in Table 1. It is clear that all analysed samples obtained from the two examined cultivars in the southern region of Peloponnese (cv. Koroneiki and cv. Mastoides), are classified in the highest quality category as “extra virgin olive oil (EVOO)” as they fullfil the demands of the current EU Regulation 2568/91 [18]. In particular, olive oils of both cultivars exhibited low mean values on their qualitative parameters. The mean free fatty acid was 0.34% for olive oils of cv Koroneiki and 0.39% for olive oils of cv. Mastoides. Respectively, the mean peroxide value for cv. Koroneiki was 7.24 meqO_2_ kg^−1^ and 6.96 meqO_2_ kg^−1^ for olive oils of cv. Mastoides. Likewise, K_232_ and K_268_ mean values were quite below the limit set by the EU Regulation 2568/91. The results depict the overall high quality of south Peloponesse olive oil production, one of the most important olive-growing regions in Greece.

### 3.2. Evaluation and Discrimination of the Two Examined Cultivars of Southern Peloponnese According to Their Fatty Acid Composition

Fatty acid composition is a crucial parameter for the quality and characterization of an olive oil [20]. Because of the fact that the fatty acid content is a fundamental parameter for the determination of the nutritional properties of olive oil, the description of a specific cultivar on the basis of their fatty acid composition is of utmost importance. As a result, many researchers have used fatty acid composition in order to group olive oils according to the origin of the cultivar [21,22,23].

In the present study, the GC-FID analysis of the 112 olive oil samples from Koroneiki and Mastoides cultivars showed their complete fatty acid composition. As shown in Table 2, all values of the thirteen fatty acids identified, were in conformity to the normal range expected for olive oil category for both cultivars. Generally, olive oils of Koroneiki cultivar had a mean value of 76.70% for the mono-unsaturated oleic acid (C18:1) compared to olive oils of Mastoides cultivar which had a mean value of 75.93% (*p* < 0.05). Moreover, olive oils of Koroneiki presented a higher concentration with respect to the poly-unsaturated linolenic acid (C18:3) with a mean value of 0.68% compared to cv. Mastoides (0.55%). On the other hand, olive oils of cv. Mastoides were characterized by a clearly higher concentration in heptadecanoic acid (C17:0) with a mean value at 0.14% and in heptadecenoic acid (C17:1) with a mean value at 0.25% compared to the olive oils of cv. Koroneiki which had almost a three-fold lower concentration, with mean values 0.05% and 0.08%, respectively. No differences were observed for the following fatty acids: myristic (C14:0), palmitic (C16:0), palmitoleic (C16:1), and linoleic acids (C18:2) as shown in Table 2.

As mentioned in the introduction, there is one relevant publication by Stefanoudaki et al. [17] where the authors examined the same cultivars in the island of Crete. They reported that olive oils of Koroneiki cultivar were characterized by lower concentrations of oleic (C18:1) and heptadecanoic acids (C17:0) and higher concentrations of linoleic (C18:2) and palmitic acids (C16:0). Those differences can be explained by the fact that apart from the olive cultivar other secondary factors, mainly environmental, (e.g., different climatic conditions such as temperature, rainfall, humidity at each growing site), have a significant effect on the composition of the fatty acid profile [24,25].

**Figure 1:**
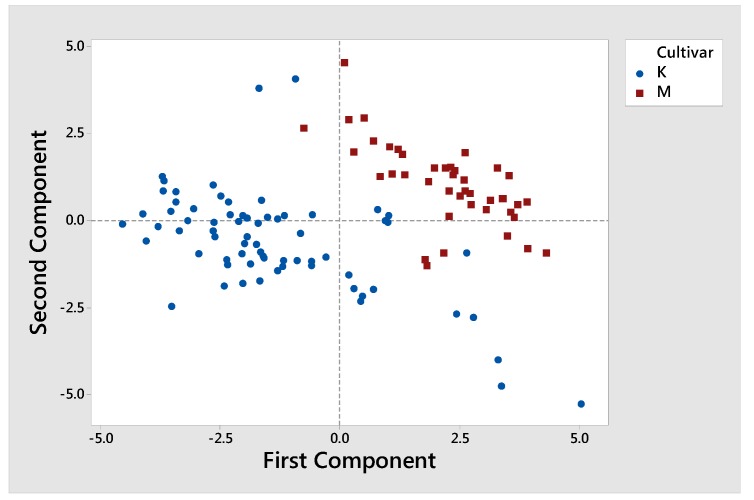
Score plot of principal component analysis (PCA) for cv. Koroneiki and cv. Mastoides obtained from olive trees in southern Peloponnese according to their fatty acid composition. K corresponds to Koroneiki olive oils (blue dots) and M to Mastoides olive oils (red dots).

As shown in Table 2, the fatty acid composition data of the 112 olive oil samples were subjected to analysis of variance. It was revealed that, apart from C14:0, C16:0, C16:1, and C18:2, substantial differences were observed between Koroneiki and Mastoides cultivars in all the rest analyzed fatty acids (*p* < 0.05). Additionally, principal component analysis (PCA) on fatty acid composition data was performed to confirm and enhance the classification according to the cultivar. PCA can be used to decrease the initial variables into a limited number of new variables (principal components) describing most of the variation in the originals. The main purpose of the key factor analysis, taken together, is to define related variables. The first two principal components are significant and explain approximately the 84% of the variation in the data. Thus, based on PCA in Figure 1 we showed the score plot of PCA for cv. Koroneiki and cv. Mastoides according to their fatty acid composition. In this case we found that most of the points for K are pointed to the left of PC1, meaning that K has large negative loadings on component 2. On the other hand, points for M are presented on the right of PC1, meaning that M has large positive loadings on component 1. The K and M regions are therefore independent of each other and the chemical properties studied are also independent and the regions are affected by them. Hence, the application of the PCA algorithm to the fatty acid data revealed a discrete separation between the two cultivars, by creating two distinctive clusters. The results are in agreement with studies by Stefanoudaki et al. where they concluded that fatty acid compositional data of Koroneiki and Mastoides cultivar showed significant potential for olive oil classification [17].

There are other studies focusing on olive oil compounds with the capability to differentiate among cultivars, highlighting that fatty acid composition data can be used as a traceability marker of the botanical origin [21,26,27,28]. For example, D’ Imperio et al. by analyzing Sicilian extra virgin olive oils from 22 cultivars found out that oleic, linoleic and palmitic fatty acids were crucial in the characterization of the olive oil cultivars [21]. Likewise, Krichene et al. determined the content of fatty acids and phenolic compounds, as well as other olive oil minor components in Tunisian olive cultivars; observing clear differences between them [28].

### 3.3. Evaluation and Discrimination of the Two Examined Cultivars From the Southern Region of Peloponnese According to Their Sterolic Profile

Olive oil is characterized by several minor components with an important nutritional impact on human health [29,30]. Phytosterols and triterpenic dialcohols are included among them and constitute the major proportion of the unsaponifiable fraction of olive oil (around 20%). Many researchers have revealed that the application of different chemometric treatments on the sterols present in olive oils or a combination of specific individual sterols with other chemical parameters can discriminate among olive cultivars [31,32,33,34,35]. For example Lukic et al. demonstrated that sterols and triterpene diols can be used as reliable indicators of variety and ripening degree among virgin olive oils from Croatia [31]. Another research group has shown that the combination of total sterol content, campesterol, stearic acid, and oxidative stability enabled the classification of olive oils according to their variety [36].

Although several studies have been conducted for Greek mono-cultivars in other regions of Greece [37,38,39,40], in the present study, the sterolic composition and content from the two monovarietal olive oils of southern Peloponnese were evaluated and compared. Table 3 lists the mean values expressed as percentage of the individual sterols and total sterols concentration of the two monocultivars. The individual sterols and total sterols content for the examined olive oil sample of Mastoides cultivar were within the established EU regulatory limits [18]. In general, Mastoides oils exhibited higher mean value for β-sitosterol (84.12%) and lower mean value for Δ-5-avenasterol (9.85%) and total erythodiol content (1.40%) compared to the relative values for Koroneiki olive oils (Table 3). In addition, higher concentration in the mean total sterols was observed in Mastoides olive oils (1219.6 mg/kg) compared to the olive oils of Koroneiki cultivar, where the mean value was 1033.3 mg/kg, very close to the regulatory set limit of 1000 mg/kg according to the EU regulation 2568/91 [41].

**Figure 2:**
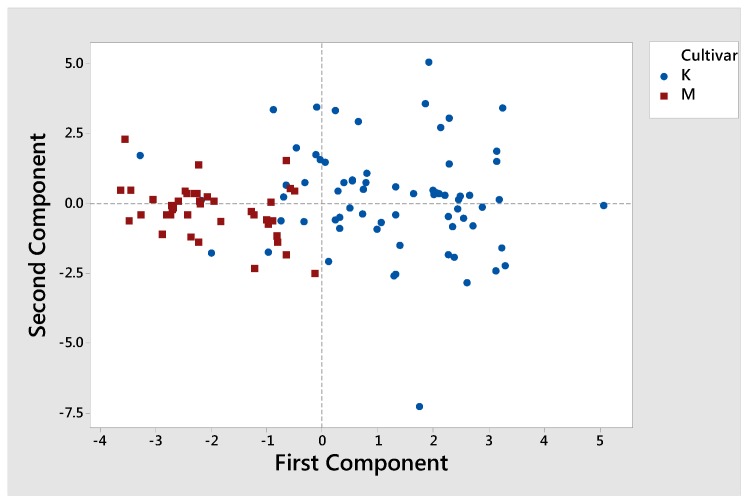
Score plot of PCA for cv. Koroneiki and cv. Mastoides according to their sterolic profile. K corresponds to Koroneiki olive oils (blue dots) and M to Mastoides olive oils (red dots).

As shown in Table 3, by comparing the two cultivars, the calculated *p*-value according to their sterolic profile, was in most cases close to 0.00 (*p* ≈ 0.00), indicating a strong botanical effect. No previous reported data is available to compare and to the best of our knowledge, it is the first time to examine the sterolic profile of cv. Mastoides. The PCA score plot of Koroneiki versus Mastoides olive oils according to their sterolic profile is presented in Figure 2. The first two principal components explain approximately the 81% of the variation in the data. It is observed that most of the K points are shown to the right of PC1, hence K has large positive loadings on component 1. On the other hand, most of the M points have large negative loadings on component 2. Thus, K and M regions are independent of each other according to their sterolic profile, permitting a clear classification of the examined monocultivars in two separated clusters. Relevant studies in Greek olive cultivars have been carried out classifying Greek olive oils according to cultivar and geographical origin, based on the composition of their volatile compounds [42], phenolic compounds and fatty acids composition [38].

**Figure 3:**
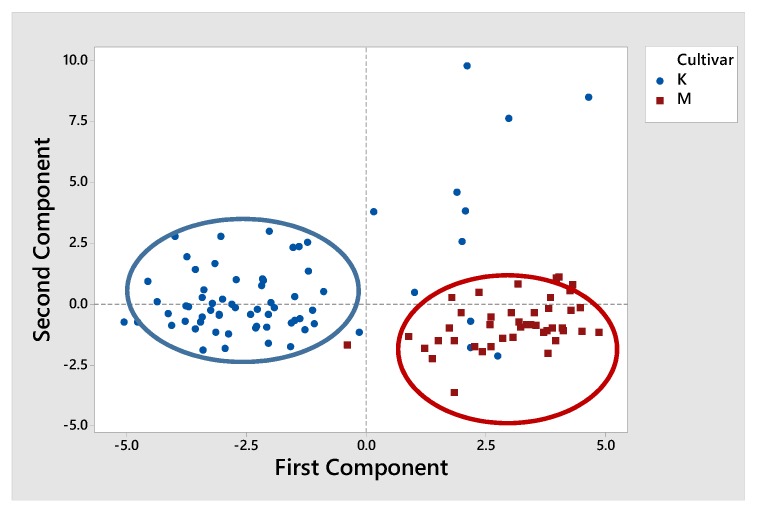
Score plot of PCA for cv. Koroneiki and cv. Mastoides olive oil samples of southern Peloponnese according to the combination of fatty acid compositional data and sterolic profile. K corresponds to Koroneiki olive oils (blue dots) and M to Mastoides olive oils (red dots).

According to many authors, chemometric tools can also be used to select the best variables to obtain satisfactory results [32,33,34,36,43,44]. As a result, a combined principal component analysis was performed using both fatty acid compositional data and individual/total sterols as variables. To simplify the method used to limit a large set of variables to a small set but holding most of the detail in the large set, PCA was applied in this case too. The first two principal components illustrate data variation of 81%. The score plot of PCA for cv. Koroneiki and cv. Mastoides according to the combination of fatty acid compositional data and sterolic profile is shown in Figure 3. In this scenario, we found that the majority of the points for K stand on the left side of PC1, and hence K has large negative loads on component 2. On the other side, most of the points for M stand on the right of PC1, thus implying M has large positive loads at component 1. Both K and M regions are therefore independent of each other according to the combination of fatty acid and sterolic profile.

Thus, it is evident to conclude that fatty acid and sterolic profile data can permit the discrimination of the examined extra virgin olive oils in south Peloponnese region in terms of olive cultivar and can be used as useful authenticity-traceability indicators.

## 4. Conclusions

In the present study we demonstrated that fatty acid compositional data and sterols have a high differentiation potential as authenticity tools. Meanwhile, analyses on other more or less exploited Greek monocultivars need to be performed in order to reveal and evaluate their quality and chemical characteristics so as to establish a national authenticity databank. Finally, the possibility of investigating other components present in olive oils and taking into account new authenticity methodologies would be useful for the comparison of different Greek monocultivars in the region.

## Figures and Tables

**Table 1:** Qualitative parameters from the two major olive cultivars of southern Peloponnese.

	cv. Koroneiki (N = 69)		cv. Mastoides (N = 43)		EEC Limit for EVOO Category
Parameter	Mean ± SD	Min–Max	Mean ± SD	Min–Max	
Free acidity (%)	0.34 ± 0.13	0.17–0.76	0.39 ± 0.13	0.15–0.77	≤0.80
Peroxide value (meqO_2_/kg)	7.24 ± 1.88	3.64–11.96	6.96 ± 2.31	2.88–14.70	≤20
K_232_	1.55 ± 0.14	1.33–2.14	1.63 ± 0.11	1.33–2.02	≤2.50
K_268_	0.13 ± 0.01	0.08–0.21	0.12 ± 0.02	0.08–0.17	≤0.22

Results are expressed as means ± standard deviation (SD). N = 112.

**Table 2:** Fatty acid profile of cv. Koroneiki and cv. Mastoides cultivated in southern Peloponnese.

	cv. Koroneiki (N = 69)		cv. Mastoidis (N = 58)		Calculated *p*-Value	EEC Limit for EVOO Category
Parameter	Mean ± SD	Min–Max	Mean ± SD	Min–Max		
Myristic C14:0 (%)	0.01 ± 0.00	0.00–0.02	0.01 ± 0.00	0.00–0.02	n.s	≤0.03
Palmitic C16:0 (%)	12.02 ± 0.74	9.54–13.56	12.29 ± 0.77	9.96–13.28	n.s	7.50–20.00
Palmitoleic C16:1 (%)	0.92 ± 0.13	0.64–1.43	0.92 ± 0.10	0.64–1.08	n.s	0.30–3.50
Heptadecanoic C17:0 (%)	0.05 ± 0.02	0.03–0.15	0.14 ± 0.02	0.08–0.17	0.00	≤0.40
Heptadecenoic C17:1 (%)	0.08 ± 0.04	0.06–0.24	0.25 ± 0.03	0.16–0.29	0.00	≤0.60
Stearic C18:0 (%)	2.53 ± 0.19	1.98–3.12	2.64 ± 0.16	2.35–2.99	0.001	0.50–5.00
Oleic C18:1 (%)	76.70 ± 1.96	70.67–81.40	75.93 ± 1.27	73.15–79.44	0.024	55.00–83.00
Linoleic C18:2 (%)	6.09 ± 1.60	4.20–12.01	6.44 ± 0.69	5.11–8.13	n.s	2.50–21.00
Linolenic C18:3 (%)	0.68 ± 0.07	0.51–0.86	0.55 ± 0.04	0.49–0.66	0.00	≤1.00
Arachidic C20:0 (%)	0.44 ± 0.03	0.33–0.50	0.39 ± 0.02	0.35–0.44	0.00	≤0.60
Eicosenoic C20:1 (%)	0.31 ± 0.02	0.27–0.35	0.27 ± 0.02	0.23–0.32	0.00	≤0.50
Behenic C22:0 (%)	0.14 ± 0.01	0.09–0.17	0.10 ± 0.01	0.07–0.12	0.00	≤0.20
Lignoceric C24:0 (%)	0.05 ± 0.00	0.03–0.08	0.04 ± 0.007	0.03–0.06	0.00	≤0.20

Results are expressed as means ± standard deviation (SD). n.s = not-significant. The statistical significance level was set at *p* < 0.05.

**Table 3:** Sterol profile of cv. Koroneiki and cv. Mastoides cultivated in southern Peloponnese.

	cv. Koroneiki (N = 69)	cv. Mastoidis (N = 43)	Calculating *p*-Value	EEC Limit for EVOO Category
Sterols and Triterpene Diols	Mean ± SD	Mean ± SD		
Cholesterol (%)	0.11 ± 0.03	0.12 ± 0.03	0.017	≤0.5
24-methylene-cholesterol %	0.32 ± 0.09	0.19 ± 0.05	0.00	
Campesterol %	3.71 ± 0.38	3.14 ± 0.16	0.00	≤4.0
Campestanol %	0.05 ± 0.03	0.04 ± 0.02	n.s	<campesterol
Stigmasterol %	0.74 ± 0.19	0.64 ± 0.18	0.01	
Chlerosterol %	0.85 ± 0.07	0.94 ± 0.07	0.00	
β-Sitosterol %	80.73 ± 3.73	84.12 ± 2.69	0.00	
Sitostanol %	0.37 ± 0.30	0.31 ± 0.08	n.s	
Δ-5-avenasterol %	12.28 ± 3.96	9.85 ± 2.66	0.001	
Δ-5, 24-stigm/dienol %	0.29 ± 0.10	0.22 ± 0.06	0.00	
Δ-7-stigmastenol %	0.19 ± 0.09	0.18 ± 0.09	n.s	≤0.5
Δ-7-avenasterol %	0.28 ± 0.11	0.22 ± 0.06	0.001	
Apparent b-Sitosterol %	94.63 ± 1.07	95.45 ± 0.29	0.00	≥93.0
Total Erythrodiol %	2.85 ± 1.25	1.40 ± 0.52	0.00	≤4.5
Total sterols (mg/kg)	1033.3 ± 150.1	1219.6 ± 109.2	0.00	≥1000

Results are expressed as means ± standard deviation (SD). n.s = not-significant. The statistical significance level was set at *p* < 0.05.
